# Prognostic value of the preoperative study of cerebrospinal fluid dynamics in Chiari malformations: a pilot study

**DOI:** 10.1007/s00701-025-06710-5

**Published:** 2025-11-21

**Authors:** Pauline Carlier, Romaric Lantonkpode, Johann Peltier, Olivier Balédent, Cyrille Capel

**Affiliations:** 1 Department of Neurosurgery, University Hospital Amiens-Picardie, 80054 Amiens, France; 2 CHIMERES UR 7516, University of Picardie Jules Verne, 80000 Amiens, France; 3 Image Processing Department Hospital University Center of Amiens Picardie, 80054 Amiens, France

**Keywords:** Phase-contrast MRI, Chiari malformation, Cerebrospinal fluid dynamics

## Abstract

**Purpose:**

In patient with Chiari type I malformations (CM1), indication for surgery can be difficult to establish. Headaches are a common complaint. Factors that predict headache relief have not been clearly identified. Several studies have aimed to examine cerebrospinal fluid (CSF) hydrodynamics in patients with CM1 by using phase-contrast MRI (pcMRI), which is currently the only non-invasive method for assessing craniospinal hydrodynamics and hemodynamics. People with CM1 present alterations in cerebrospinal fluid (CSF) and cerebral blood dynamics. The objective of the present study was to identify hydrodynamic criteria that are predictive of positive clinical outcome (headache relief) after posterior fossa decompression surgery in patient with CM1.

**Method:**

41 patients who underwent posterior fossa decompression surgery at Amiens-Picardie University Hospital (Amiens, France) between 2016 and 2021 were retrospectively included. We used preoperative pcMRI to analyze CSF dynamics. Stroke volumes of cerebrospinal fluid were quantified at the aqueduct of Sylvius (SV_aqu_), subarachnoid spaces near to C2-C3 (SV_C2C3_) vertebral junction, prepontine cisterns, foramen magnum, and brainstem. CSF pulsatility was analyzed in relation to whether patients reported postoperative headache relief. Statistical analyses were based on Student's t-test.

**Results:**

12 patients reported headache relief. The mean SV_aqu_ was significantly higher in patients with headache relief than in those without relief (65 and 32.13µL/CC, p ≤ 0.05). The mean SV_C2-C3_ was significantly lower in patients with headache relief than in patients without relief (484.58 and 612.94µL/CC, p ≤ 0.05). The two groups of patients did not differ significantly in terms of the area of the narrowest part of the aqueduct of Sylvius or the Evans index.

**Conclusion:**

SVaqu may have prognostic value for headache relief following surgery for CM1. Further investigation is warranted. This association is likely related to the recruitment of intraventricular pulsatility, which may help regulate potential intracranial pressure changes. Notably, this pulsatility does not appear to be linked to morphological features.

## Introduction

Chiari malformation type 1 (CM1) is radiologically defined in adults by caudal displacement of the cerebellar tonsils of more than 5 mm below the foramen magnum, typically measured relative to the McRae line, which connects the basion to the opisthion [4]. The clinical presentation is variable and often non-specific, including headache, neck pain, and sensory [4]. Some patients report otorhinolaryngological symptoms, spinal cord involvement, bulbar involvement, or visual disturbances [22]. The most commonly reported symptom by patients is headache [32] conventionally described as pulsatile, exacerbated by exertion or coughing, and radiating to the vertex. CM1-related headache has a major impact on the patient’s quality of life and is often resistant to drug treatment [5]. Some patients also experience so-called atypical headaches, which are similarly refractory to medical therapy and not always attributable to differential diagnoses CM1 [5].

When symptoms markedly impair quality of life, surgical treatment may be considered. The standard procedure involves posterior fossa decompression, with or without duraplasty [11]. The surgical technique does not appear to predict clinical outcomes; however, posterior fossa decompression with duraplasty seems to offer better symptom relief [9, 10, 14]. A major challenge in CM1 surgery is selecting patients whose symptoms are likely to improve postoperatively [9, 10, 14]. It is important to note that this procedure is associated with a non-negligible morbidity rate. Recent large-scale studies report an overall postoperative complication rate of approximately 11–12%, with major complications—such as cerebrospinal fluid leaks requiring reoperation or neurological deterioration—occurring in 1.8% of cases [18]. Headache is the primary indication for surgery in Chiari malformation type I, yet recent studies report that approximately 24% of patients with preoperative headache do not experience postoperative improvement, with 5–7% even reporting persistent or worsened symptoms [24]. Establishing surgical indications is therefore complex, due to the current lack of clearly identified predictive factors for postoperative headache relief in CM1. Only occipital throbbing headaches radiating toward the vertex appear to be associated with favourable surgical outcomes [5], while other headache types have an uncertain prognosis. These findings provide guidance for clinical decision-making but fall short of offering a reliable prediction of treatment efficacy.

The aetiology of CM1 remains unclear [24]. Some studies have shown that symptom severity does not correlate with the degree of cerebellar tonsillar herniation [15]. Moreover, clinical outcomes do not appear to be directly related to morphological variables [23]. We therefore chose to investigate cerebrospinal fluid (CSF) hydrodynamics in an effort to better understand the genesis of CM1 and its associated symptoms [1, 8]. During systole, there is a sharp increase in cerebral arterial blood flow, resulting in a sudden influx of arterial blood into the cranial cavity[7, 19]. This inflow is fully compensated by an outflow of venous blood [3, 20]. However, arterial and venous flows are not synchronized throughout the cardiac cycle (CC), leading to a transient increase in intracranial vascular volume during systole. In healthy individuals, subarachnoid CSF is rapidly displaced into the perimedullary subarachnoid spaces during systole, thereby almost instantaneously compensating for fluctuations in intracranial vascular volume [3, 20]. The pulsatility of the subarachnoid CSF contribute to overall intracranial compliance. Thus, CSF oscillations at the level of the foramen magnum during the CC play a key role in regulating intracranial pressure.

Patients with CM1 exhibit alterations in CSF and cerebral blood flow dynamics [7, 12, 19, 26, 27]. The downward herniation of the cerebellar tonsils reduces the cross-sectional area of the subarachnoid spaces at the level of the foramen magnum, thereby increasing resistance to CSF flow. In some cases, pulsatile forces are transmitted to the cerebellar tonsils and neuraxis, which may represent a characteristic marker of CM1 [7, 19, 27]. These hydrodynamic disturbances may be associated with specific clinical manifestations of CM1. Phase-contrast magnetic resonance imaging (PC-MRI) remains the only non-invasive technique capable of assessing CSF and blood flow dynamics throughout the CC.

Headaches represent one of the most subjective symptoms and are therefore among the most difficult to treat. Basing a surgical indication solely on this symptom is challenging, especially when considering the risk–benefit balance. For this reason, our study focuses exclusively on the postoperative improvement of this specific symptom.

This preliminary study aimed to assess whether the preoperative evaluation of CSF dynamics could serve as a predictor of postoperative headache relief in patients with CM1.

## Material and methods

### Patients

All patients diagnosed with Chiari malformation type I (CM1) who underwent posterior decompression surgery at Amiens-Picardie University Hospital (Amiens, France) between 2016 and 2021 were retrospectively included in this study. All patients received posterior fossa decompression with or without duraplasty. The diagnosis of CM1 was confirmed in all cases based on morphological MRI criteria. Patient characteristics were recorded during the initial diagnostic consultation. In this cohort, 27 patients presented with typical headaches, while 14 patients exhibited atypical headache profiles. Postoperative headache relief was defined as a reduction of more than two points on a pain visual analogue scale, accompanied by an improvement in quality of life. Quality of life was assessed based on patients’ self-reports, specifically whether they were able to resume daily activities that had initially been limited. No other symptoms were considered in the current analysis.

Surgical indications were therefore primarily based on the presence of pulsatile headaches, which are classically associated with CM1, and/or other concomitant symptoms including torticollis, medullary tract signs, sleep apnea, vertigo, gait instability, nystagmus, visual blurring, tinnitus, vestibular dysfunction, swallowing difficulties, dyspnea, hydrocephalus, or syringomyelia. The decision to operate was thus guided by a comprehensive clinical and radiological evaluation rather than headache symptoms alone. This approach may explain why some patients without typical headache presentations nevertheless benefited from surgical intervention.

### Imaging

#### MRI acquisition

Each patient was diagnosed with CM1 based on morphological MRI criteria. At the Amiens-Picardie University Hospital, all patients diagnosed with CM1 undergo additional pcMRI sequences prior to any surgical treatment. Brain MRI examinations were performed on a 3 Tesla system (Philips Achieva), with imaging parameters detailed in Table 1. During pcMRI acquisition, the velocity encoding was set at 10 cm/s to measure the stroke volume (SV) of CSF passing through the aqueduct of Sylvius and to 5 cm/s for acquisitions in other planes.

The pcMRI measurements were synchronized with the patient's heart rate through retrospective plethysmographic gating; allowing velocity changes to be measured at 32 time points throughout the CC. The pcMRI images were analyzed using Flow® software (version 2.0, 2018) developed internally at Amiens-Picardie University Hospital.

#### pcMRI post-processing

**Fig. 1:**
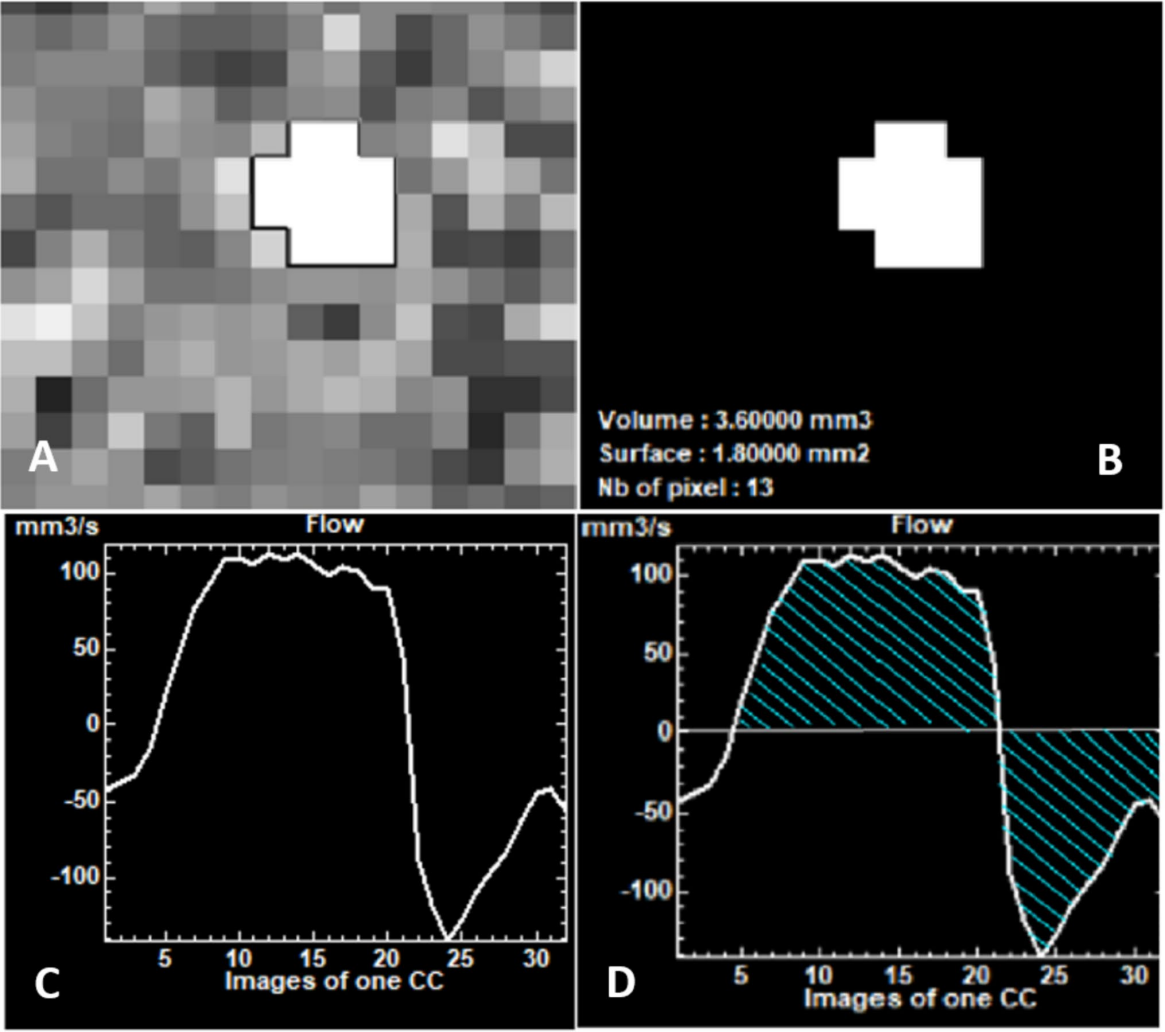
**A**) Phase-contrast MRI (PC-MRI) data of the aqueduct of Sylvius. **B**) Semi-automatic segmentation of the aqueduct of Sylvius using Flow software (developed in-house at Amiens-Picardie University Hospital) to isolate the region of interest for measuring the cerebrospinal fluid (CSF) stroke volume (SV) passing through the aqueduct during the cardiac cycle. **C**) CSF flow curve through the aqueduct plane over the cardiac cycle, generated using Flow software and the semi-automatic segmentation described above. D) Integration of the CSF flow curve, yielding the stroke volume of CSF passing through the aqueduct of Sylvius during the cardiac cycle

**Fig. 2:**
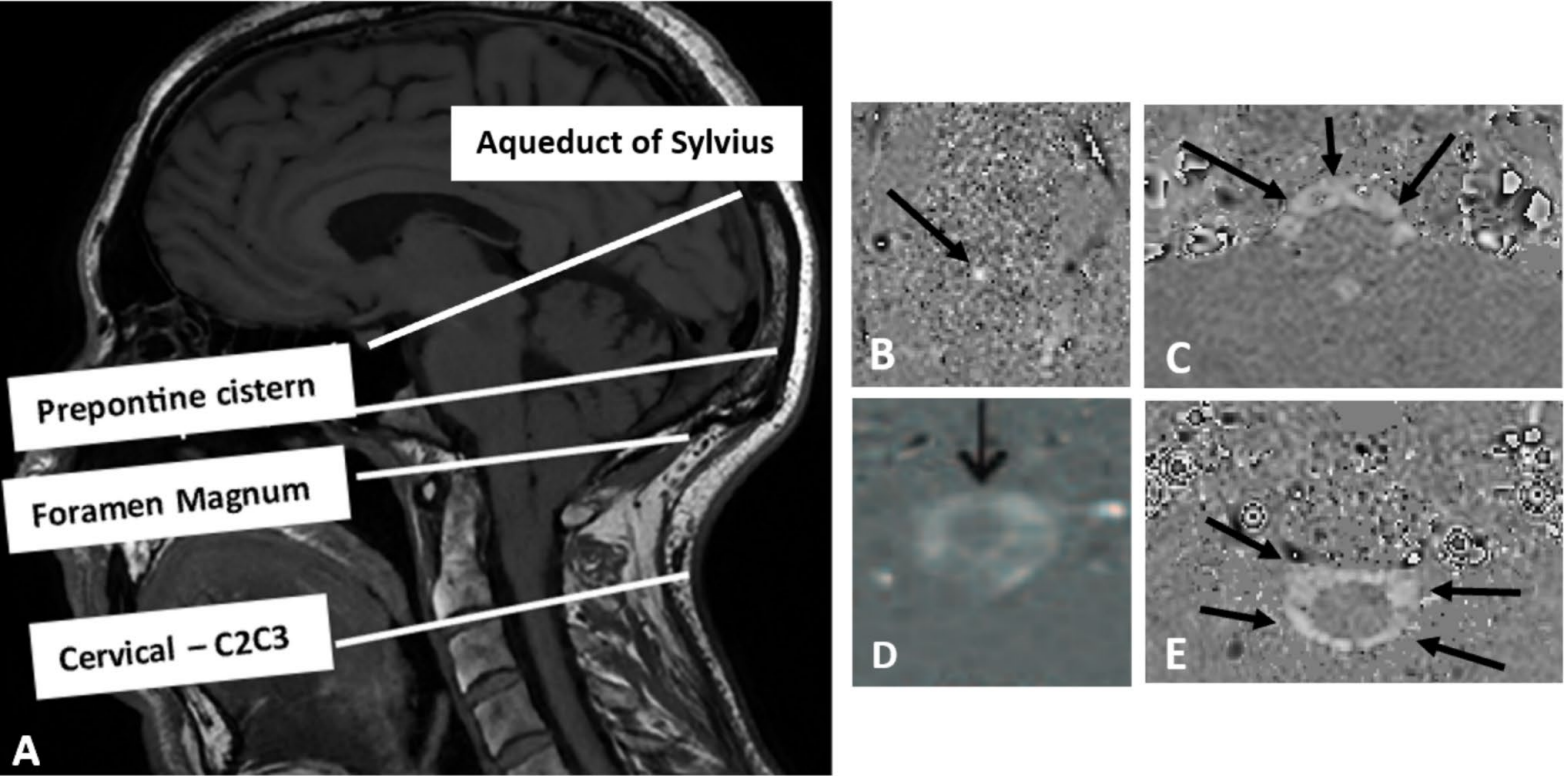
**A**) Morphological T1-weighted MRI (sagittal sections) illustrating the regions of interest where cerebrospinal fluid (CSF) stroke volume (SV) was measured during the cardiac cycle: the aqueduct of Sylvius, and the subarachnoid spaces adjacent to the prepontine cistern, the foramen magnum, and the C2-C3 vertebral junction. B–E) Corresponding phase-contrast MRI (PC-MRI) data for **B**) the aqueduct of Sylvius, **C**) the prepontine cistern, **D**) the foramen magnum, and **E**) the C2-C3 junction

Using the preoperative pcMRI data, we reconstructed voxel-wise CSF velocity curve. For each predefined imaging plane, the region of interest was segmented semi-automatically using Flow® software (Fig. 1). Each voxel in the area of interest corresponded to a CSF velocity during a CC. The resulting velocity curve represented the CSF flow through the region of interest (Fig. 1). This flow curve was subsequently integrated to calculate the stroke volume (SV) of CSF passing through the region during the cardiac cycle (Fig. 1). Applying this method to each plane and region of interest, we quantified CSF stroke volumes at multiple precise anatomical sites throughout the cardiac cycle[7]: the mesencephalic aqueduct (SVaqu), the cervical subarachnoid spaces near the C2-C3 vertebral junction (SVC2C3), the prepontine cistern (SVppc), the foramen magnum (SVfm), and the cerebellar tonsils adjacent to the foramen magnum (SVtonsils) (Fig. 2).

#### Measurement of the area of the aqueduct of Sylvius

The cross-sectional area (mm^2^) of the aqueduct of Sylvius was measured on axial slices obtained from preoperative 3D T2 fast field echo acquisitions. This sequence offers superior contrast between tissue and fluid signals. On sagittal slices, the location where the aqueduct of Sylvius is narrowest was identified and reconstructed in the axial plane. The region of interest was subsequently delineated manually.

#### Measurement of the Evans index

For each patient, the Evans index was calculated from preoperative morphological MRI data, using axial sections from fluid-attenuated inversion recovery (FLAIR) sequences. The Evans index is defined as the ratio of the maximum width of the frontal horns to the maximum biparietal diameter of the cranium and serves as a marker of ventriculomegaly [2]. All Evans index measurements were performed manually by a single examiner.

###  Statistics

Statistical analyses were performed using Student’s t-test. Data normality was assessed with the Kolmogorov–Smirnov test. Statistical significance was set at p ≤ 0.05. For intergroup comparisons of the aqueduct of Sylvius area and Evans index, a non-parametric Mann–Whitney test was applied due to the non-normal distribution of these variables.

### Ethics

The study protocol was approved by the local institutional review board (CPP Nord Ouest II, Amiens, France; reference: HYDROAQU-C PI2024_843_0028). In accordance with French legislation regarding retrospective observational studies of clinical practice, patient consent was waived.

## Results

### Clinical data

A total of 41 patients (37 women and 4 men) were included (Table 1). The mean age was 39 years. At the postoperative follow-up visit, 12 patients reported headache relief, while 29 reported no relief. There were no significant differences between the relief and no-relief groups in terms of demographic characteristics or preoperative symptom profiles (Table 1). The postoperative improvement rate, defined by a Chicago Chiari Outcome Scale (CCOS) which is a validated clinical tool used to assess postoperative outcomes in patients treated for Chiari malformation type I. It evaluates four domains: pain symptoms, non-pain symptoms, functionality, and complications. Each domain is scored on a scale from 1 to 4, with higher scores indicating better outcomes. The total CCOS score ranges from 4 to 16, allowing a standardized and comprehensive assessment of clinical improvement following surgical decompression. A score between 13 and 16, was 92.7% (38 of 41 patients).

**Table 1:** Demographic characteristics of the study population, according to the presence or absence of headache relief after surgery

Population	Headache relieved	Headache not relieved
Number of patients	12	29
Sex ratio (M/F)	3/9	3/26
Mean [range] age (years)	45 [30–66]	39 [19–74]

27 patients presented with typical headaches and 14 patients with atypical headaches. Among the improved population, 4 patients (33%) of patients had atypical headaches, while 8 patients (65%) of patients in the non-improved group presented with typical headaches.

### Hydrodynamic parameters

Preoperative pcMRI analysis revealed that the mean stroke volume at the aqueduct of Sylvius (SVaqu) was significantly higher in patients who experienced headache relief compared to those without relief (65 µL/CC vs. 32.13 µL/CC, respectively; p ≤ 0.05) (Table 2). Conversely, the mean stroke volume at the C2-C3 cervical subarachnoid space (SVC2-C3) was significantly lower in patients with headache relief than in those without postoperative relief (484.58 µL/CC vs. 612.94 µL/CC, respectively; p ≤ 0.05).

**Table 2:** Preoperative mean ± SD CSF SVs over the CC in patients with vs. without headache relief. * Indicates a statistically significant difference (p ≤ 0.05) based on Student’s t-test, following confirmation of data normality by the Kolmogorov–Smirnov test

SV (µL per CC)	Headache relieved (n = 12)	Headache not relieved (n = 29)	p
SV_aqu_	65 ± 45	32 ± 24	0.03*
SV_ppc_	334 ± 262	410 ± 172	0.32
SV_fm_	436 ± 301	504 ± 225	0.62
SV_nevrax_	248 ± 155	157 ± 109	0.19
SV_C2-C3_	485 ± 163	613 ± 166	0.03*

**Fig. 4:**
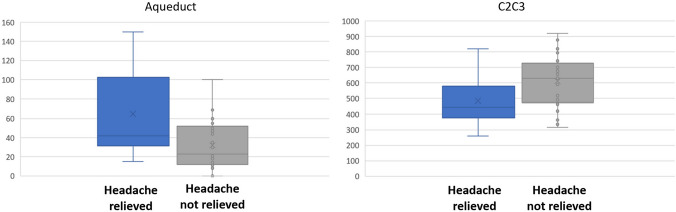
Distribution of the CSF SV_aqu_ and SV_C2C3_ (measured in µL per CC) in patients with vs. without headache relief after surgery

Although the postoperative cerebrospinal fluid SV ranges overlapped between the two patient groups, an SVaqu value exceeding 100 µL per cardiac cycle was observed exclusively in patients who experienced headache relief after surgery, whereas values below 18 µL per CC were only found in patients without headache relief postoperatively (Fig. 4). Similarly, an SVC2-C3 value below 300 µL per CC was observed solely in patients with headache relief, while values above 800 µL per CC were only present in those without postoperative headache relief.

### Morphological data

The two groups of patients did not differ significantly in terms of the area of the narrowest part of the aqueduct of Sylvius or the Evans index (p > 0.05).

## Discussion

This study found that preoperative CSF flow parameters differ between patients with and without postoperative headache relief following posterior fossa decompression for CM1. Specifically, higher aqueductal stroke volumes and lower cervical subarachnoid stroke volumes were associated with symptom improvement, while morphological MRI features did not differ significantly between groups.

Our current findings could potentially pave the way for preoperative assessment of CM1 using phase contrast MRI. Indeed, high intraventricular CSF pulsatility prior to surgery could have prognostic value for the improvement of postoperative headaches, particularly atypical headaches. This discovery could help surgeons select patients who may benefit from surgery or, conversely, recommend drug treatment alone, in order to avoid unnecessary surgery for headaches, but also to offer surgery even in cases of atypical headaches.

### Headaches

According to some studies, patients presenting with typical headaches tend to have better postoperative outcomes than those with atypical headache patterns. In our series, this trend was also observed, with higher improvement rates among patients with typical headaches. Nonetheless, several individuals with atypical headaches—particularly when associated with other CM1-related symptoms—also experienced clinical benefits following decompressive surgery, even when headaches were not the predominant complaint.

This observation aligns with previous findings. Thunstedt et al. reported that Valsalva-induced (cough) headaches were more responsive to surgery than migraine-like or tension-type patterns [28]. Similarly, a recent meta-analysis found that typical headache types were associated with higher improvement rates following decompression compared to atypical forms [16].

These results suggest that headache phenotype may influence surgical prognosis. However, the presence of atypical headache does not exclude potential benefit from surgery, especially when supported by additional clinical or radiological findings. Prognostic expectations should therefore remain cautious and individualized. Our study included both patients with typical headaches (pulsatile, exacerbated by exertion or coughing, radiating to the vertex) and those with atypical presentations (all other types). In our series, 27 patients presented with typical headaches and 14 patients with atypical headaches. Among the improved population, 33% of patients had atypical headaches, while 65% of patients in the non-improved group presented with typical headaches. Therefore, some patients with atypical headaches did experience improvement. Consequently, our study highlights a clear benefit of decompression surgery in selected cases of patients presenting with atypical headaches. However, some patients with atypical headaches experience clinical relief after surgery, and so it is difficult to base selection for surgery purely on the patient’s subjective description of headache; objective variables (such as those based on pcMRI data) are needed. The preoperative profile of symptoms other than headache did not appear to have prognostic value because it was similar in patients with vs. without postoperative headache relief.

This study focuses on the postoperative course of headache evolution; however, the indication for surgery was not based solely on this symptom. All operated patients presenting with headaches were included, and their postoperative symptom evolution was documented. Nonetheless, the decision to operate was also guided by the presence of other clinical signs, such as pseudobulbar syndrome, involvement of lower cranial nerves, or other cranial nerve deficits.

Some patients underwent surgery primarily based on headache presentation, among whom 92% experienced headache improvement. Investigating headache improvement is particularly relevant, as some patients exhibited atypical headache patterns that nevertheless responded favourably to surgical intervention.

### Hydrodynamics and prognostic assessment

Few studies have investigated the relationship between craniospinal hydrodynamics and clinical symptoms. Several have reported an association between the presence of symptoms and abnormal cerebrospinal fluid flow on cine phase-contrast MRI, although these findings have not established abnormal cerebrospinal fluid flow on cine phase-contrast MRI as definitive diagnostic markers [21].

However, these studies focused solely on the maximum flow velocity of the CSF or blood. The volume displaced depends directly in the pressure gradient [13]. Hence, an analysis of the displaced volume appears to be entirely appropriate. Some research have already described the value of pcMRI for predicting postoperative clinical improvement in patients with CM1 [12, 21, 26]. McGirt et al. [21] has been demonstrated that normal preoperative CSF flow in the hindbrain, as assessed by pcMRI, constitutes an independent risk factor for failure to achieve postoperative headache relief in patients with CM1. pcMRI may therefore prove useful in identifying Chiari type 1 malformation patients at higher risk of poor response to surgical decompression [21]. A high preoperative SVaqu appears to predict postoperative headache relief. This may reflect the recruitment of ventricular compliance, as CM1-associated herniation of the cerebellar tonsils likely alters subarachnoid compliance by increasing flow resistance in this region.

Our findings also suggest that changes in cervical subarachnoid CSF dynamics may be related to postoperative headache prognosis. Consistent with previous studies, a postoperative decrease in CSF dynamics—specifically, a reduction of at least 20% in SV_C2C3_—is associated with headache relief [26]. Nevertheless, this significant difference appears to have less clinical relevance than the analysis of intraventricular CSF dynamics. Indeed, the distribution of CSF hydrodynamic profiles (Fig. 4) demonstrates that most patients exhibited similar profiles. This similarity may be attributable to differing hemodynamic patterns, as CSF pulsatility is directly influenced by variations in intracranial vascular volume [20].

### Aqueduct area and Evans index

Evans’ index is not frequently used in CM1 without hydrocephalus. Nevertheless, it is an index reflecting ventricular volume [6]. Intraventricular CSF dynamics may be related to ventricular volume [17, 29]. We therefore used this index to evaluate the various factors influencing intraventricular CSF dynamics. According to Poiseuille’s law, fluid flow depends on the pressure gradient, flow area, and fluid viscosity. Intraventricular CSF dynamics arise from variations in the pressure gradient between the 3rd and 4th ventricles. Since CSF viscosity remains constant, and no significant difference was observed in the cross-sectional area of the midbrain aqueduct between patients with and without headache relief, the increase in intraventricular CSF dynamics appears to be attributable to variations in the pressure gradient across the aqueduct of Sylvius. Measuring this pressure gradient could therefore be valuable to determine its association with postoperative headache relief. This gradient may underlie the observed elevated intraventricular pulsatility.

The results of several studies of patients with normal pressure hydrocephalus have demonstrated that the SV at the aqueduct of Sylvius depends essentially on the ventricular volume [25]. In our study, we observed a higher preoperative CSF SV in the group of patients who experienced headache relief. However, this increased SV was not associated with ventricular enlargement, as the two patient groups did not differ significantly in terms of the Evans index, a reliable marker of ventriculomegaly. Moreover, recent studies suggest that integrating preoperative assessments of cerebrospinal fluid hydrodynamics with morphological analysis of the fourth ventricle outlet enhances the predictive accuracy of postoperative clinical outcomes in patients undergoing decompression surgery CM1 [30]. An increase in ventricular pulsatility, as evidenced in our study by the elevated SV of the aqueduct of Sylvius in patients with postoperative improvement, does not appear to be associated with an increase in ventricular volume. Regardless of the preoperative ventricular volume, ventricular pulsatility increases, suggesting that pulsatility is independent of ventricular size.

In our study, we focused on CSF dynamics by measuring stroke volume at the aqueduct, without assessing craniocervical junction or tonsillar motion. This approach differs from most studies, which often rely on flow velocity rather than stroke volume. Our results suggest that aqueductal stroke volume may provide additional, useful information on CSF flow in CM1 [31]. Recent literature primarily relies on CSF flow velocity, rather than stroke volume, for prognostication in CM1. Increased foramen magnum flow velocities post-decompression seems correlated with better clinical outcomes, whereas baseline symptoms had no prognostic value and peak diastolic velocity in the aqueduct significantly predicted postoperative improvement [30]. In contrast, our work focuses exclusively on stroke volume measures, offering valuable insight that complements velocity-based approaches.

### Study limitations

Firstly, our study included a small number of patients; therefore, further studies with larger populations are necessary to confirm these findings. Secondly, as our study was retrospective, prospective studies incorporating headache-specific score assessments are warranted. Thirdly, our analyses were limited to CSF dynamics, which are secondary to variations in intracranial arteriovenous volume. Consequently, investigations focusing on arteriovenous variables may provide additional prognostic insights into postoperative clinical improvements. Future research could evaluate both variations in CSF volumes and changes in arterial and venous volume dynamics during the CC. Moreover, breathing influences hemodynamics throughout the CC; thus, studying respiratory variations in this context would be of interest.

## Conclusion

Preoperative elevated CSF flow, particularly between the ventricles and the spinal subarachnoid space, may be associated with postoperative headache relief following posterior fossa decompression in patients with CM1. This alteration in CSF flow could reflect broader hydrodynamic changes occurring throughout the craniospinal axis.

## Data Availability

No datasets were generated or analysed during the current study.
